# Lichen-Derived Compounds and Extracts as Biologically Active Substances with Anticancer and Neuroprotective Properties

**DOI:** 10.3390/ph14121293

**Published:** 2021-12-10

**Authors:** Elżbieta Studzińska-Sroka, Aleksandra Majchrzak-Celińska, Przemysław Zalewski, Dominik Szwajgier, Ewa Baranowska-Wójcik, Barbara Kaproń, Tomasz Plech, Marcin Żarowski, Judyta Cielecka-Piontek

**Affiliations:** 1 Department of Pharmacognosy, Poznan University of Medical Sciences, Święcickiego 4, 60-781 Poznan, Poland; pzalewski@ump.edu.pl (P.Z.); jpiontek@ump.edu.pl (J.C.-P.); 2 Department of Pharmaceutical Biochemistry, Poznan University of Medical Sciences, Święcickiego 4, 60-781 Poznan, Poland; majchrzakcelinska@ump.edu.pl; 3 Department of Biotechnology, Microbiology and Human Nutrition, University of Life Sciences in Lublin, Skromna 8, 20-704 Lublin, Poland; dominik.szwajgier@up.lublin.pl (D.S.); ewa.baranowska@up.lublin.pl (E.B.-W.); 4 Department of Clinical Genetics, Medical University of Lublin, Radziwiłłowska 11, 20-080 Lublin, Poland; barbara.kapron@umlub.pl; 5 Department of Pharmacology, Medical University of Lublin, Chodźki 4a, 20-093 Lublin, Poland; tomasz.plech@umlub.pl; 6 Department of Developmental Neurology, Poznan University of Medical Sciences, Przybyszewski 49, 60-355 Poznan, Poland; zarowski@ump.edu.pl

**Keywords:** (−)-usnic acid, evernic acid, salazinic acid, secondary metabolites, lichen extracts, biological activity

## Abstract

Lichens are a source of chemical compounds with valuable biological properties, structurally predisposed to penetration into the central nervous system (CNS). Hence, our research aimed to examine the biological potential of lipophilic extracts of *Parmelia sulcata*, *Evernia prunastri*, *Cladonia uncialis*, and their major secondary metabolites, in the context of searching for new therapies for CNS diseases, mainly glioblastoma multiforme (GBM). The extracts selected for the study were standardized for their content of salazinic acid, evernic acid, and (−)-usnic acid, respectively. The extracts and lichen metabolites were evaluated in terms of their anti-tumor activity, i.e., cytotoxicity against A-172 and T98G cell lines and anti-IDO1, IDO2, TDO activity, their anti-inflammatory properties exerted by anti-COX-2 and anti-hyaluronidase activity, antioxidant activity, and anti-acetylcholinesterase and anti-butyrylcholinesterase activity. The results of this study indicate that lichen-derived compounds and extracts exert significant cytotoxicity against GBM cells, inhibit the kynurenine pathway enzymes, and have anti-inflammatory properties and weak antioxidant and anti-cholinesterase properties. Moreover, evernic acid and (−)-usnic acid were shown to be able to cross the blood-brain barrier. These results demonstrate that lichen-derived extracts and compounds, especially (−)-usnic acid, can be regarded as prototypes of pharmacologically active compounds within the CNS, especially suitable for the treatment of GBM.

## 1. Introduction

Secondary metabolites of lichens are polyphenolic compounds, constituting a group of natural substances with unique chemical structures and interesting biological properties. Phenolic compounds are effective against different neoplasms, which are one of the most important medical problems. Central nervous system (CNS) tumors are especially challenging, with glioblastoma multiforme (GBM) being one of the most deadly cancers. A growing body of evidence shows that natural bioactive molecules may serve well as an alternative approach to the treatment and control of GBM [1]. This prompted us to undertake further research on lichen-derived substances as important novel remedies useful in GBM therapy.

The research on lichen-derived compounds has recently been intensified, leading to the acquisition of data on their various mechanisms resulting in anticancer activity [2], as well as their anti-inflammatory, antioxidant, and neuroprotective properties [3]. Lichens grow in all continents, but *Parmelia sulcata (Parmeliaceae)*, *Evernia prunastri (Parmeliaceae)*, and *Cladonia uncialis (Cladoniaceae)* are species especially abundant in the northern hemisphere [4]. Their biological potential is attributed largely to lipophilic phenolic compounds, namely salazinic acid, evernic acid, and (−) - usnic acid, respectively [5,6]. The cytotoxic properties of salazinic acid and *P. sulcata* extracts, as well as evernic acid and *E. prunastri* lipophilic extracts, have been confirmed on various cancer cell lines [7,8,9,10]. Moreover, moderate antioxidant activity of salazinic acid [11] and *P. sulcata* extracts [10,12] has been reported. Evidence also exists for neuroprotective and anti-inflammatory properties of evernic acid, which can especially be important in the context of Parkinson’s disease [13,14]. The derivatives of dibenzofurans, including usnic acids (right and left-handed isomer) are known for their strong anticancer properties [15]. Among other research, their activity against tumors developing in the CNS (neuroblastoma, GBM) [16,17,18] was also reported. The literature indicates the anti-inflammatory potential and the anti-neurodegenerative effect [14] of these compounds. The right-handed isomer is more often studied, while data on the left-handed isoform are limited. There are also no studies on the anti-tumor activity of *C. uncialis*.

Current literature also indicates that lichen-derived compounds, as well as their synthetic derivatives, can enhance the effects of currently used anticancer drugs. For instance, the co-treatment of lobarstin, a secondary metabolite isolated from *Stereocaulon alpinum,* enhanced toxicity of temozolomide when used in GBM T98G cells [19]. In another study, the ketamine derivatives of (+)-usnic acid had significant cytotoxicity against human GBM-astrocytoma cell line U87MG, and a novel *N*-heterocyclic derivative of (+)-usnic acid was found to be even more active than temozolomide [20]. These findings justify further studies exploring the possible applications of lichenochemicals in brain tumor treatment.

In addition to the lichen-derived compounds, the biological potential of lichen-derived extracts has largely remained unexplored. Lichen extracts are a source of a plethora of compounds including pulvinic acid derivatives, terpenes, carotenoids, depsides, depsidones, depsones, anthraquinones, and xanthones [21]. Such a high diversity of compounds, often acting synergistically, makes the extracts very attractive for investigation. Moreover, there is growing evidence that crude lichen extracts often have greater in vitro or/and in vivo activity as compared to pure compounds [22].

**Figure 1:**
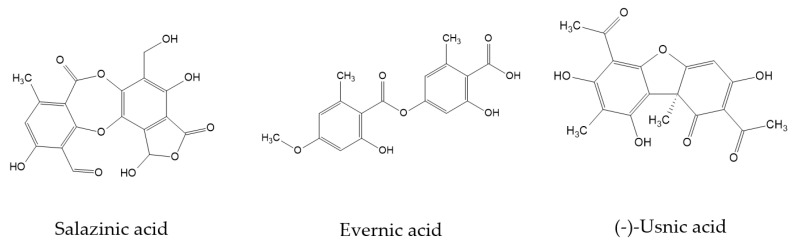
The chemical structures of lichen-derived substances—salazinic acid, evernic acid and (−)-usnic acid—investigated in this study.

Our previous study indicated the anticancer and neuroprotective potential of physodic acid and the acetone extract from *Hypogymnia physodes* [23], encouraging us to undertake further work aimed at examining the biological potential of selected lichen-derived extracts and their dominant secondary metabolites in the context of their potential application in GBM treatment and the protection of the brain tissue. Thus, in this study we evaluated the cytotoxic, anti-inflammatory, antioxidant, and anticholinergic properties of lichen acetone extracts from *P. sulcata, E. prunastri,* and *C. uncialis* as well as their most important components, salazinic acid, evernic acid and (−)-usnic acid (Figure 1), respectively. Using the in vitro PAMPA-BBB model, the penetration of the tested lichen compounds through the blood-brain barrier (BBB) was also determined.

## 2. Results

### 2.1. Phytochemicals Analysis

#### 2.1.1. Quantitative Analysis of Extracts’ Components

**Figure 2:**
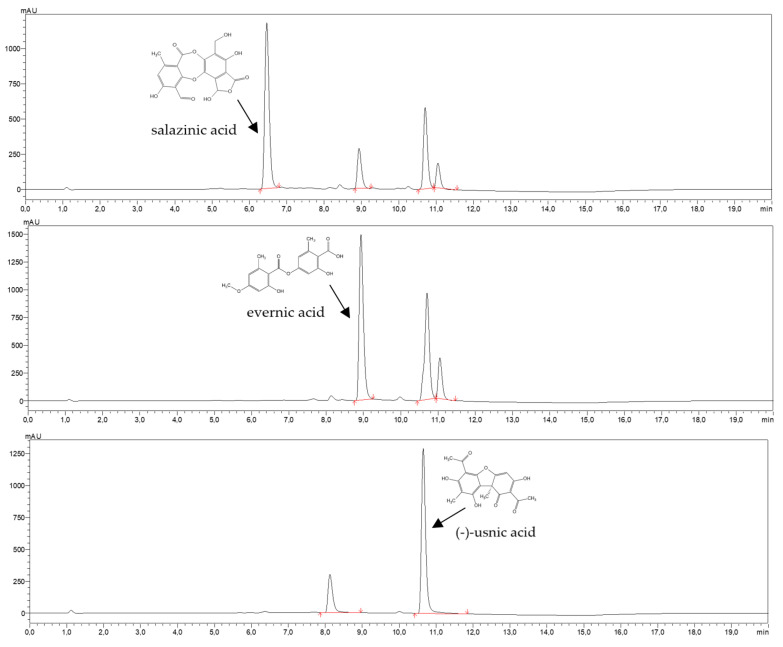
The chromatograms of acetone extract from *P. sulcata, E. prunastri,* and *C. uncialis*, showing the identified compounds, i.e., salazinic acid, evernic acid, and (−)-usnic acid, respectively. The compounds were characterised by t_R_ = 6.5 min, 8.9 min, and 10.6 min, respectively.

The content of the species-specific compounds in the lichen acetone extracts was determined using HPLC analysis. The best separation of the *P.* *sulcata, E. prunastri,* and *C. uncialis* acetone extracts was obtained with gradient elution (acetonitrile and 0.5% formic acid) on 5 μm core–shell particles. The components of the lichen extracts were separated in less than 11 min. As presented in Figure 2, the method was selective for salazinic acid (t_R_ = 6.5 min), evernic acid (t_R_ = 8.9 min), and (−)-usnic acid (t_R_ = 10.6 min).

The HPLC analysis revealed that the acetone extracts of *P. sulcata* contained 23.05% of salazinic acid, *E. prunastri* extracts contained 66.63% of evernic acid, while *C. uncialis* extracts contained 30.65% of (−)-usnic acid.

#### 2.1.2. Total Polyphenols Content

The total polyphenols content was determined in the lichen extracts using the spectrophotometric Folin–Ciocalteau method. The tested acetone extracts (*P. sulcata, E. prunastri*, *C. uncialis*) were characterized by a different content of phenolic compounds. The highest content of the phenolic compounds was measured for *E. prunastri* extract (276.95 ± 3.87 mg GAE/g of extract), as compared to *P. sulcata* and *C. uncialis* extracts, which demonstrated lower content of polyphenols (130.5 ± 0.3 mg GAE/g of extract and 71.0 ± 0.3 mg GAE/g of extract, respectively).

### 2.2. Biological Activity

#### 2.2.1. Anti-Tumor Activity

##### Cytotoxic Activity against GBM Cells

**Figure 3:**
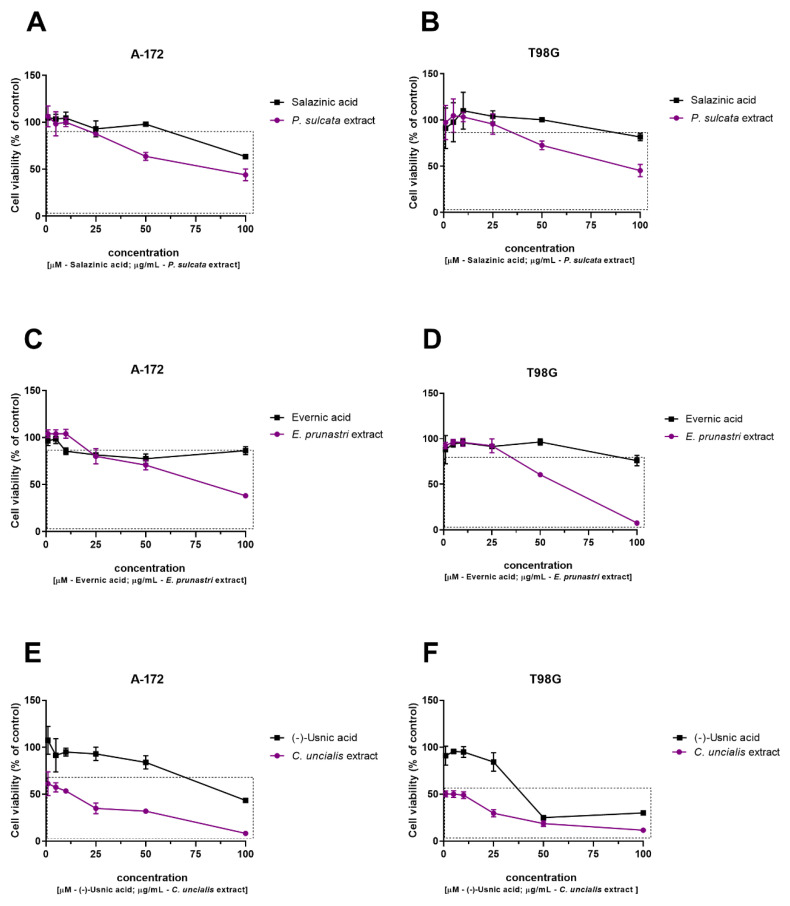
The cytotoxicity evaluation of salazinic acid and *P. sulcata* acetone extract in A-172 cell line (**A**) and T98G cell line (**B**), of evernic acid and *E. prunastri* acetone extract in A-172 cell line (**C**) and T98G cell line (**D**), and of (−)-usnic acid and *C. uncialis* acetone extract in A-172 cell line (**E**) and T98G cell line (**F**), based on 48 h MTT test. DMSO-treated cells (control) were assigned as 100% cell viability. Statistically significant difference in cell viability between the cells exposed to the analyzed lichen-derived compound or extract as compared to the control cells is indicated with a rectangle. The mean values ± SEM from three independent experiments with four measurements per assay are presented.

The viability assay showed the dose-dependent cytotoxicity of the tested lichen-derived compounds and extracts in regard to GBM cells. Generally, both A-172 and T98G cells reacted in a similar manner to the treatments (Figure 3). Salazinic acid reduced cell viability only at the highest tested concentration (100 µM), while *P. sulcata* extract diminished the percentage of living cells at 50 µg/mL and 100 µg/mL concentration in both cell lines (also 25 µg/mL concentration reached statistical significance in the A-172 cell line). Evernic acid reduced A-172 cell viability at 10 µM concentration; in the T98G cell line, however, it was only mildly cytotoxic—only the highest tested concentration led to ~ 20% reduction in cell viability. *E. prunastri* extract significantly reduced A-172 cell viability at 25 µg/mL concentration, but the highest tested concentration still allowed the survival of more than one fourth of the seeded cell population. In contrast, in the T98G cell line, 50 µg/mL *E. prunastri* extract decreased viability to 60.50 ± 0.71%, but 100 µg/mL concentration was completely cytotoxic. As far as (−)-usnic acid is concerned, all the tested concentrations up to 100 µg/mL in A-172 and 50 µg/mL in the T98G cell line were generally not cytotoxic. In regard to the *C. uncialis* extract, it significantly reduced cell viability even at 1 µg/mL, while the highest tested concentration of this extract led to almost complete cell death, i.e., only 8.33 ± 1.15% and 11.67 ± 0.58% of A-172 and T98G cells, respectively, were still alive and metabolically active. The IC_50_ values of all the analyzed lichen-derived compounds and extracts are presented in Table 1.

##### Inhibition of the Kynurenine Pathway Enzymes: Indoleamine 2,3-Dioxygenases 1 (IDO1), Indoleamine 2,3-Dioxygenases 2 (IDO2), and Tryptophan 2,3-Dioxygenase (TDO)

The kynurenine pathway plays a role in the development of GBM and is regarded as a possible molecular target for GBM treatment [24]. Thus, our next goal was to analyze if the lichen-derived compounds and extracts possess inhibitory properties related to kynurenine pathway enzymes, indoleamine 2,3-dioxygenases 1 (IDO1), indoleamine 2,3-dioxygenases 2 (IDO2), and tryptophan 2,3-dioxygenase (TDO). Our results show that out of the investigated lichen-derived compounds, only salazinic acid did not exhibit any inhibition effect against IDO1, IDO2, and TDO enzymes (Table 2). The other lichen-derived compounds and extracts, similarly to epacadostat (the reference drug with IC_50_ = 17.4 ± 1.1 nM), turned out to be IDO1 selective inhibitors. Evernic acid and (−)-usnic acid, assayed in a fixed concentration of 100 µg/mL, displayed ability to reduce the activity of IDO1 by 32.84 ± 1.93% and 21.62 ± 0.85%, respectively. The most effective inhibitory activity was shown by *C. uncialis* extract, that inhibited IDO1 by 54.82 ± 3.51%. The extracts obtained from *E. prunastri* and *P. sulcata* reduced the activity of the tested enzyme by 43.06 ± 1.97% and 20.47 ± 1.23%, respectively (Table 2).

#### 2.2.2. Anti-Inflammatory Activity

##### Inhibition of Cyclooxygenase-2 (COX-2)

Cyclooxygenase-2 (COX-2), the enzyme involved in both initiation and resolution of inflammation [25], has been implicated in tumorigenesis and progression of GBM [26]. Overexpression of COX2 appears also during natural or pathological aging of the brain [27]. The results of our study indicate that all the tested lichen-derived compounds and extracts exerted anti-COX-2 effects. As shown in Table 3, the most potent anti-COX-2 activity was shown by *P. sulcata* extract (65.9 ± 4.1%) and salazinic acid (60.3 ± 3.0%). (−)-Usnic acid and evernic acid were also characterized by strong anti-inflammatory activity, inhibiting COX-2 to 59.3 ± 3.5% and 50.7 ± 2.1%, respectively. The anti-COX-2 activity of the other analyzed extracts, namely *E. prunastri* and *C. uncialis* extracts were weaker, though still noticeable.

##### Anti-Hyaluronidase Activity

**Figure 4:**
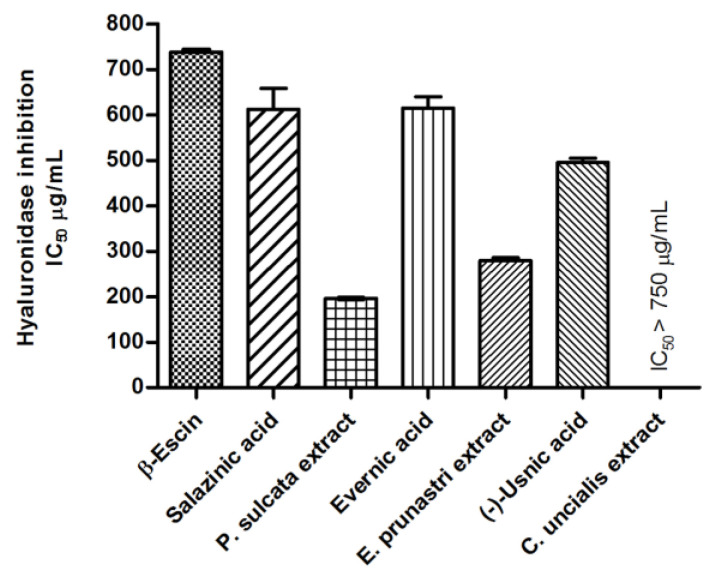
Inhibition of hyaluronidase by lichen-derived substances and extracts as well as by β-escin (served as a standard in this assay). Results are presented as IC_50_ mean values (mg/mL) ± SEM (five measurements; *n* = 5 for lichen-derived compounds; and *n* = 6 for β-escin) obtained in two independent experiments.

Hyaluronidase is an enzyme responsible for maintaining the homeostasis of hyaluronan in the extracellular matrix. Upregulation of this enzyme activity is observed in chronic inflammatory conditions [28]. In addition, hyaluronidase, responsible for generating smaller fragments of hyaluronidase (HA), may be crucial for the correct functioning of the CNS [29]. Therefore, we determined the effect of lichen-derived compounds and extracts on hyaluronidase activity. The results presented in Figure 4 show that in the tested concentration range, lichen-derived compounds and extracts, excluding *C. uncialis* extract (IC_50_ > 0.75 mg/mL), demonstrate the ability to inhibit hyaluronidase (Figure 4). In fact, the inhibitory properties of salazinic acid and evernic acid were similar to those obtained for β-escin, used as a standard. In addition, we noticed the pronounced difference between the extracts’ activity and the activity of pure compounds. The inhibitory potential was more pronounced for the examined extracts.

#### 2.2.3. Impact on Reactive Oxygen Species (ROS) Homeostasis

The disruption of CNS homeostasis may be caused by oxidative stress [30]. However, in cancer cells ROS are able to trigger programmed cell death and ROS generation is an important mechanism of chemo- and radio-therapy [31]. Therefore, it is important to determine how lichen-derived compounds and extracts influence ROS homeostasis. In this context, assessment of the antioxidant activity of *P. sulcata, E. prunastri, C. uncialis* acetone extracts, and salazinic acid, evernic acid (−)-usnic acid isolated from these lichens, was undertaken using two different spectroscopic methods. The well-known DPPH method assesses the free radical scavenging ability of a sample in vitro, while the CUPRAC method shows the ability of the tested substances to reduce cations of metals. As displayed in Table 4, only *P. sulcata* and *E. prunastri* extracts were able to scavenge the DPPH radical and reduce Cu^2+^ ions measured in the assumed concentration range. In the DPPH test, the *P. sulcata* extract was more active than the *E. prunastri* extract. The CUPRAC method showed that *E. prunastri* extract reduced metal ions more strongly than *P. sulcata* extract. It is worth mentioning that the detected activity of *E. prunastri* was only 3–4 times lower than resveratrol, which is considered as a potent antioxidant. None of the tested lichen-derived compounds showed antioxidant activity in the performed experimental models and concentrations.

##### Effect on Antioxidant Enzyme Activity

Superoxide dismutase (SOD), glutathione reductase (GR), and glutathione peroxidase (GPx) are the main endogenous enzymatic defense systems of human cells [32]. Therefore, we checked the influence of the lichen-derived compounds and extracts on SOD, GR, and GPx using in vitro spectroscopic methods. In regard to SOD, the present study showed that both lichen-derived compounds and extracts have the capacity to inhibit the activity of this enzyme (Table 5). The highest inhibitory activity was detected for *E. prunastri* extract, as it inhibited more than half of SOD activity (53.4% ± 2.4%) (Table 5). *P. sulcata* extract and its major constituent salazinic acid, as well as evernic acid and (−)-usnic acid, inhibited SOD activity by ~20%. The weakest, although still noticeable SOD inhibitory activity was exerted by *C. uncialis* extract (12.8% ± 1.0%).

The inhibitory effect of lichen substances on GR and GPx enzymes was also determined in our study. The results show that the most potent inhibitory effect was demonstrated by *E. prunastri* extract, which inhibited both GR and GPx. Interestingly GPx was inhibited only in 20.0 ± 2.1% by evernic acid, contrasted to 92.4 ± 4.3% by the *E. prunastri* extract, evernic acid. Both enzymes (GP and GPx) were also inhibited by (−) - usnic acid, but more weakly than by the *E. prunastri* extract. Quite potent inhibition against GPx was also presented by the PS extract, which did not inhibit the GR at all. Salazinic acid and *C. uncialis* extract showed no activity (Table 6).

#### 2.2.4. Anticholinergic Activity

Cholinesterases are a group of enzymes responsible by the hydrolysis of acetylcholine, playing a fundamental role in neurosynaptic communication. Increased activity of these enzymes has also been noted in brain tumors [33]. Thus, to investigate the anticholinesterase effect (anti-AChE and anti-BChE) of *P. sulcata, E. prunastri, C. uncialis* and isolates of salazinic acid, evernic acid and (−)-usnic acid, the modified Elman’s method was used. Our results indicated the diversified activity of the tested extracts and compounds. The extract of *C. uncialis* only, weakly inhibited AChE. BChE, an enzyme with lower substrate specificity [34], was inhibited by most of the tested substances (Table 7). The greatest inhibition of BChE was shown by evernic acid.

### 2.3. Permeability through the Blood-Brain-Barrier (PAMPA-BBB)

The presence of an active compound at the site of its action is essential for achieving a biological effect. Thus, using the Parallel Artificial Membrane Permeability Assay for the Blood-Brain Barrier (PAMPA-BBB) we evaluated if lichen-derived compounds, both used as pure substances and as the components of extracts, can cross the BBB and reach the CNS. The results of our analysis revealed that the permeability of the tested compounds varied significantly. For (−)-usnic acid and evernic acid, whether it was permeated from the extract or as a pure compound, the calculated *Pe* proved their high permeability (*Pe* > 1.5 × 10^−6^ cm/s) [35,36]. We noted that (−)-usnic acid penetrated extremely strongly compared to the other tested compounds. This is indicated by the very high *Pe* index achieved after 1 h incubation time. Salazinic acid, the depsidone from *P. sulcata*, was characterized by the very low *Pe* coefficient (*Pe* < 0.5 × 10^−6^ cm/s). Data analysis also showed that the degree of permeation is similar, whether the compound is permeated from the extracts or as a pure substance (Table 8).

### 2.4. Summary of Biological Potential of Lichen-Derived Compounds and Extracts

**Figure 5:**
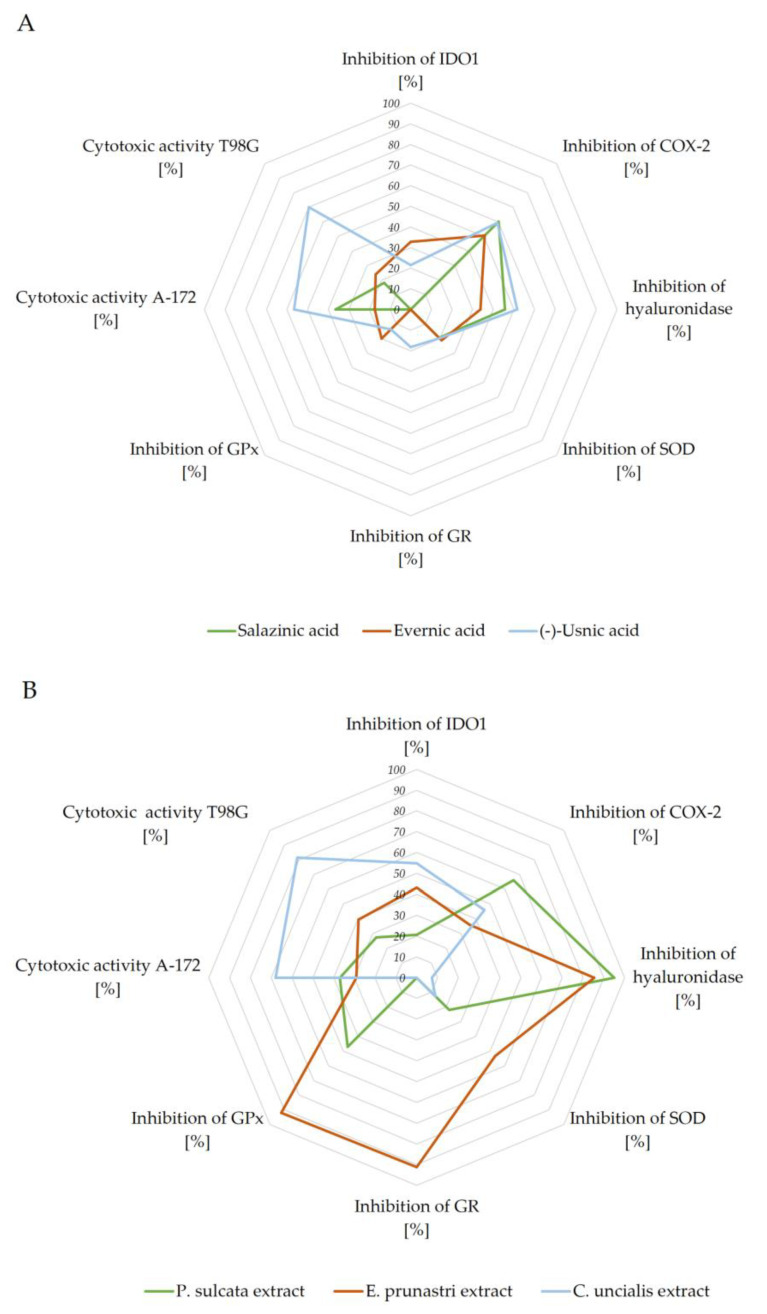
The biological activity of lichen-derived compounds and extracts: compounds (**A**) and extracts (**B**), expressed by the surface of area, taking into account the measured biological properties expressed in %. The graphs were made for the concentrations: inhibition of IDO1 100.0 µg/mL (**A**,**B**); inhibition of COX-2 250.0 µg/mL (**A**,**B**); inhibition of hyaluronidase 500.0 µg/mL (**A**,**B**); inhibition of SOD 537.6 µg/mL (**A**,**B**); inhibition of GR 444.4 µg/mL (**A**,**B**); inhibition of GPx 243.9 µg/mL (**A**,**B**); cytotoxicity expressed as % of cell death: A-172 100 µM (**A**), 50 µg/mL (**B**), and T98G 100 µM (**A**), 50 µg/mL (**B**).

To summarize the results obtained, we presented the data as a star diagram (Figure 5). This chart allowed us to assess the total biological potential in terms of the assessed directions of activity. The highest activity among the tested compounds was characterized by (−)-usnic acid. The other two compounds were less active. Evernic acid was involved in all directions of activity presented in the graph (cytotoxic, anti-inflammatory, and antioxidant inhibitory activity). These properties were admittedly a bit weaker than those demonstrated by salazinic acid, but more diverse. Salazinic acid was, however, more potent as an anti-inflammatory agent, but it was not active in regard to IDO1 inhibition.

The analysis of the biological potential of the extracts showed that *C. uncialis* extract had the highest cytotoxic activity and the lowest anti-inflammatory and antioxidant enzyme-inhibiting activity. On the other hand, *E. prunastri* extract most strongly inhibited the antioxidant enzymes. Still, it had the weakest anti-inflammatory effect, and its cytotoxic effect on cancer cells was comparable to that of *P. sulcata* extract. However, its higher IDO1 inhibitory capacity suggests a greater antitumor potency as compared to *P. sulcata* extract. In fact, the latter exhibited the weakest properties in regard to anticancer properties.

The analysis of the charts also allowed us to observe the differences in the potency between pure compounds and the extracts. The presented figure shows that the compounds tested in the same concentration as the extracts (see Figure 5 caption) had lower activity as compared to the extracts from which they originated. The potency of the extracts was sometimes even several times higher. It is important to note that the lichen-derived extracts contain a plethora of compounds, which acting together shift the extract’s properties from the ones exerted by its main components. For instance, *C. uncialis* extract and (−)-usnic acid differed in their anti-inflammatory properties, while in case of *E. prunastri* extract, its ability to inhibit antioxidant enzymes was found to be significantly enhanced due to the presence of components besides evernic acid. In turn, the extract of *P. sulcata* had high anti-inflammatory activity, especially expressed by the ability to inhibit the activity of hyaluronidase. This may indicate the synergism of the action of the substances present.

The cytotoxic activity of the extracts was slightly increased or remained at a comparable level as compared to the pure compounds. What is more, considering the amount of active substance in the tested extracts, especially for *C. uncialis* and *P. sulcata* extracts, the cytotoxic effect on GBM cells was more pronounced than for (−)-usnic and salazinic acid, respectively. To conclude, our data suggest that the lichen-derived extracts are more promising anti-GBM agents, as compared to pure lichen-derived substances.

## 3. Discussion

Lichens are an interesting group of organisms that draw attention due to the production of secondary metabolites with multidirectional biological properties [2,14,15,37]. We therefore selected three species of lichens, *P. sulcata, E. prunastri,* and *C. uncialis* for our research. Our phytochemical analysis revealed that the analyzed extracts of *P. sulcata, E. prunastri,* and *C. uncialis* are rich in polyphenolic compounds. Similar observations were reported by other research groups [22,38]. The HPLC method applied in order to determine the content of the dominant active compounds detected the major secondary metabolites; in *P. sulcata* extract it was salazinic acid, in *E. prunastri* extract it was evernic acid, and in *C. uncialis* it was (−)-usnic acid. The active compounds were abundant in the lichen-derived extracts. In this context, e.g. evernic acid constituted 66.63% of the *E. prunastri* extract, while salazinic acid constituted 23.05% of *P. sulcata* extract. The high content of polyphenols in non-polar lichen extracts was also confirmed by others. According to Manojlović et al. [10], salazinic acid was the dominant compound in acetone extract from *P. sulcata*. Other works indicated that evernic acid and (−)-usnic acid were the important components of acetone extracts of *E. prunastri* [6,38] and of *C. uncialis* [22,39], respectively.

Despite the growing evidence of the anticancer potential of lichen-derived substances and extracts, not much is known about their ability to influence brain tumor growth. Brain tumors are a serious therapeutic problem, the largest of which is GBM, being still one of the main challenges in clinical oncology [40]. Efforts are constantly being made to find more efficient anti-GBM therapy, as the overall survival of GBM patients rarely exceeds 15 months [41]. Since beneficial anticancer activity associated with lichen-derived compounds and extracts were previously reported in respect of various cancer cell lines, we hypothesized that *P. sulcata, E. prunastri* and *C. uncialis* may also decrease the viability of GBM cells. Our research confirmed the well-established role of (−)-usnic acid as a substance that decreases cancer cell viability [15]. In temozolomide-sensitive A-172 and temozolomide-resistant T98G cell line models, (−)-usnic acid was the dominant cytotoxic compound as compared to the other two lichen-derived compounds, namely, the depsidone, salazinic acid and the depside, evernic acid. Interestingly, *C. uncialis* extract even at a concentration of 1 µM, was able to significantly reduce GBM cell viability. The potential mechanisms responsible for the observed cytotoxicity can be related to pro-apoptotic properties of usnic acid, as reported for various cell line models, including colorectal adenocarcinoma (CaCo2), rhabdomyosarcoma (RD), cervical carcinoma (Hep2C), and hepatocellular carcinoma (HepG2) [2]. Data concerning the effect of usnic acid on GBM cells are, however, scarce. Only recently, the cytotoxic and genotoxic effects of (+)-usnic acid on GBM cell line U87MG cells were confirmed by Emsen et al. [17].

So far over 70 different metabolites have been identified and characterized in *E. prunastri*, and evernic acid is regarded as one of its major secondary metabolites [42]. In our study, evernic acid was cytotoxic to the A-172 cell line in a broad range of concentrations (10–100 µM); however, the cytotoxic effect was moderate – the viability dropped to ~80%, as compared to the vehicle-treated control. In the T98G cell line, the cytotoxic effects of evernic acid were observed only for the highest tested concentration (100 µM). Shcherbakova et al. found that the U-87 GBM cell line was sensitive to evernic acid, but also to *E. prunastri* extract [43]. In our study, the treatment with *E. prunastri* extract dose-dependently reduced the number of living cells. Interestingly, the temozolomide-resistant cell line, T98G, was more prone to *E. prunastri* induced cell death, as compared to the temozolomide-sensitive A-172 cell line. Furthermore, the report of Kosanić and coworkers also demonstrates that acetone extracts of *E. prunastri* and *Pseudevernia furfuraceae* possess anticancer activity against human melanoma FemX and human colon carcinoma LS174 cell lines [9].

To the best of our knowledge, *P. sulcata*, and salazinic acid were not yet tested in a model of human GBM cells; here we report their moderate cytotoxicity. Ari et al. [44] demonstrated cytotoxic effects of *P. sulcata* methanolic extracts in the C6 rat GBM cell line—cell viability significantly decreased after high doses (50 and 100 μg/mL) of the extract. In this study, *P. sulcata* extract was slightly more cytotoxic to C6 and to liver cancer Hep3B cell lines, as compared to human lung cancer A549 and PC3 cells [44].

Equally important as the cytotoxic activity of the analyzed compounds/extracts on cancer cells, is the lack of toxicity against healthy cells. Current data supports the safe profile of lichen-derived compounds and extracts in regard to normal astrocytes or neurons, supporting their neuroprotective properties [45]. For instance, in a recent study [46], evernic acid protected primary cultured neurons against 1-methyl-4-phenylpyridium (MPP+)-induced cell death, mitochondrial dysfunction, and oxidative stress, and effectively reduced MPP+-induced astroglial activation by inhibiting the NF-κB pathway. Moreover, evernic acid ameliorated 1-methyl-4-phenyl-1,2,3,6,-tetrahydropyridine-induced motor dysfunction, dopaminergic neuronal loss, and neuroinflammation in the nigrostriatal pathway in C57BL/6 mice. The neuroprotective effects of usnic acid were also reported in an acute mouse model of Parkinson’s disease. It was found that 1-methyl-4-phenyl-1,2,3,6,-tetrahydropyridine-induced motor dysfunction and neuronal loss were ameliorated in the usnic acid-treated mice *versus* vehicle-treated controls [46].

Apart from the cytotoxic properties that the investigated lichen-derived compounds and extracts exerted upon cancer cells, our study also revealed that they also possess desirable inhibitory effects on indoleamine-2,3-dioxygenase 1 (IDO1). IDO1, together with IDO2 and TDO (tryptophan-2,3-dioxygenase), are responsible for the conversion of l-tryptophan into l-kynurenine (Kyn), which is the rate-limiting step of the kynurenine pathway. The downstream metabolites of the Kyn pathway play significant role in the formation of an immunosuppressive environment, due to the negative regulation of T-cell responses [47]. It has been observed that the excessive degradation of tryptophan or the accumulation of its metabolites reduces the ability of the immune system to destroy tumor cells and also increases the progression of brain tumors. Comprehensive studies on cancer patients proved that expression and activity of IDO1 is strongly correlated with pathological grades of glioma [48]. Moreover, overexpression of IDO1 correlates with poor prognosis in patients with glioma. Therefore, IDO1 has become an attractive target in the treatment of GBM. Our results showed for the first time that *C. uncialis* and *E. prunastri* extracts, in concentrations of 100 µg/mL, are able to inhibit IDO1 by 54.82 ± 3.51% and 43.06 ± 1.97%, respectively. Interestingly, similarly to the results of the cytotoxicity study, both *C. uncialis* and *E.prunastri* extracts exhibited stronger IDO1 inhibitory properties than the pure compounds, used in the equivalent concentrations. Therefore, it should be assumed that it is not the dominant compound which is mostly responsible for the inhibitory activity towards IDO, and the final biological effect of the extracts depends on the action of other extract components. This is also evidenced by the analysis of the activity of *P. sulcata* extract, for which the IDO inhibitory activity was determined as 20% enzyme inhibition, while pure salazinic acid was not active. If these results could be extrapolated from the preclinical to clinical settings, the use of the above-mentioned extracts or their secondary metabolites (evernic and usnic acids) could be considered as a supplementary (add-on) therapy for GBM patients. This hypothesis needs further verification in cell-based assay for IDO inhibition and during in vivo experiments. However, previous studies proved that combination of IDO1 inhibitors with chemo- or immunotherapy led to an increased response rate when compared to classical therapies. The use of IDO1 inhibitors as add-on therapy can also be effective in inhibiting IDO-induced angiogenesis and thus reducing tumor growth and metastatic potential [49]. Due to the fact that IDO activation plays a pivotal role in the processes of cancer initiation, progression and metastasis, lichen extracts could possibly be used as dietary supplements in chemoprevention of cancer as well.

Because the inflammatory process accompanies many pathological conditions, and reveals itself in cancers and degenerative diseases of the brain tissue, we decided to examine also the anti-inflammatory potential of the tested lichen-derived compounds and extracts. Most brain tumors, including malignant glioma, show high COX-2 expression [50]. The activity of this enzyme was found to be correlated with the rate of GBM cell proliferation [51], with GBM grade [52], and poor prognosis [50]. Moreover, it has been shown that COX-2 activity in GBM adversely affects epilepsy accompanying the disease [50]. Hence, one of the therapeutic targets to control the development of GBM and its accompanying symptoms may be COX-2. The literature indicates the anti-inflammatory properties of lichen compounds [37], including activity towards COX-2 [23,53,54]. The results of our study indicate that all the tested lichen-derived compounds and extracts showed the ability to inhibit COX-2. However, taking into account the content of test substances in the extracts calculated based on HPLC analysis, the activity of the extracts was comparable to the anti-COX-2 properties of tested compounds. The strongest inhibition of COX-2 was exerted by *P. sulcata* extract and its component—salazinic acid. The detected COX-2 inhibitory effect was higher than that of acetylsalicylic acid. The literature confirms the anti-inflammatory properties of salazinic and evernic acids, which strongly inhibited microsomal prostaglandin E2-1 synthase [53], as well as of (+)-usnic acid, which inhibited the synthesis of leukotriene B4 (LTB4) [55]. The obtained data indicate that the substances we tested, both in pure form and in extracts, can attenuate the inflammatory response induced by COX-2, which can be beneficial both in the context of brain tumors as well as neurodegenerative diseases.

Hyaluronic acid is a component of the brain’s extracellular matrix. Hyaluronan particle size is associated with invasion of GBM cells and frequently-occurring therapeutic resistance [56]. Low molecular weight hyaluronan molecules, formed as the end product of degradation by hyaluronidase, are often associated with increased invasion and accelerated tumor growth [57] and increased cancer proliferation and cell adhesion [58]. From the point of view of reducing the invasiveness of the tumor, inhibition of the degradation of hyaluronic acid by the enzyme is an advantageous feature. Moreover, the activity of hyaluronidase may induce inflammation accompanying pathological changes in the brain tissue [28]. Thus, in this study, we wanted to verify whether the selected compounds and lichen extracts may affect the activity of hyaluronidase. Our results indicate that among the tested substances, those from two species, *P. sulcata* and *E. prunastri,* provide compounds capable of inhibiting hyaluronidase activity. In these two cases, in the concentration range used, the extract was more potent than the pure compound, and the activity was higher than that of β-escin, used as a standard [59]. Such results suggest that, apart from the tested compounds, the inhibition of the enzyme is also influenced by other substances contained in the extracts. On the other hand, salazinic acid, evernic acid, and (−)-usnic acid activity were similar to the reference. The anti-hyaluronidase activity of (−)-usnic acid was similar to that presented in recent literature [60].

ROS are involved in different signaling pathways to control cellular stability [61]. Their excess is related to neurodegeneration, but in relation to cancer treatment it is beneficial, as most anticancer therapies rely on ROS-induced cell death. Thus, it is important to know whether lichen-derived compounds and extracts possess antioxidant, or rather pro-oxidant properties. The results of our study show that *P. sulcata*, *E. prunastri* and *C. uncialis* extracts and their major secondary metabolites are characterized by very limited antioxidant properties. This is in line with other research data showing that extracts from *E. prunastri* and *P. sulcata* have little ability to scavenge ROS [12,38]. However, in our study the observed ability of these extracts to reduce copper ions was noticeable. The reducing properties of *E. prunastri* were only three times lower than that of resveratrol, used as a standard [62]. Other researchers have studied the antioxidant effect of some lichen-derived substances. In a study by Kosanić et al. [9], *E. prunastri* extract and evernic acid showed weak free radical scavenging activity in the DPPH test, compared to the standard. Salazinic acid and usnic acid were, in turn, assessed as strong antioxidants [10,63]. Our analyzes did not confirm these results. It is worth mentioning that it has been reported that the level of antioxidant activity may vary depending on the lipophilicity of the reaction environment [64]. These observations may partially explain the discrepancies in the results.

Superoxide dismutase (SOD), glutathione reductase (GR), and glutathione peroxidase (GPx) are the main endogenous enzymatic defense systems of human cells. They play important roles in neuroprotection [65] but, in the case of an already developed CNS tumor, it was observed that a high level of these enzymes correlates with a high degree of malignancy of neoplastic cells, a shorter period of disease progression, and the development of drug resistance [66]. The literature indicates that lowering the activity of SOD, GR, and GPx may increase the effectiveness of the treatment [67]. The results of our study indicate that *P. sulcata, E. prunastri,* and *C. uncialis* and their major secondary metabolites inhibit the SOD enzyme, while the strongest activity was characterized by *E. prunastri* extract (53.4%). Salazinic acid, evernic acid, and usnic acid inhibited the enzyme with similar effectiveness of about 20%. GPx was most strongly inhibited by *E. prunastri* extract (92.4 ± 4.3%), while the enzyme was not inhibited by salazinic acid and *C. uncialis* extract. Therefore, probably both salazinic acid and (−)-usnic acid do not participate in the antioxidant activity of the examined extracts. GR was only inhibited by *E. prunastri* extract (91.1 ± 7.2%) and (−)-usnic acid (18.2 ± 2.2%). These results may suggest that lichen-derived compounds and extracts, in particular *E. prunastri* extract and (−)-usnic acid, may enhance the effectiveness of GBM therapies.

Another therapeutic target in CNS diseases is acetylcholine-metabolizing cholinesterases. As their activity is increased in neurodegenerative diseases, inhibition of these enzymes positively affects patients with degenerative changes in the CNS, e.g., Alzheimer’s disease. Increased AChE and BChE activity was also observed in brain tumors, including GBM [68]. Our study found only a small or no inhibitory effect of the studied lichen-derived compounds and extracts on the activity of cholinesterases. Only the extract of *C. uncialis* showed the activity against AChE, compared to the reference substance. A similarly low inhibitory activity of lichens secondary metabolites against both AChE and BChE was confirmed in our other studies [23] as well as by other authors [69].

Due to the blood-brain barrier (BBB), which limits the penetration of most anticancer drugs into the CNS, standard GBM treatment is limited to surgical resection, followed by radiotherapy in combination with temozolomide [70]. Therefore, it is important to know if a molecule with therapeutic properties can penetrate into the CNS. One of the methods of obtaining such data is the PAMPA-BBB analysis, which allows collecting preliminary data on whether the studied molecule can show the ability to diffuse passively through the BBB. Very little is known about the ability of lichen-derived compounds and extracts to penetrate into the CNS. Our most recent study demonstrated that physodic acid is characterized by a high permeability coefficient, meaning it can reach the CNS via passive diffusion [23]. In this study we showed that evernic acid and especially (−)-usnic acid, reaching a very high *Pe* value after 1 h of incubation, can also penetrate the BBB well. Thus, taking into account our observations on the penetration and strong cytotoxicity of (−)-usnic acid, we can suppose that the ability of this compound to penetrate the CNS is high enough to have a cytotoxic effect on neoplastic cells. It has to be noted that evernic and usnic acids can penetrate via the BBB both as single substances and as active ingredients of the extracts. In contrast, our study showed that salazinic acid is incapable of penetration through the tested type of biological barrier, regardless of whether it was a component of the extract or a pure compound. The analysed lichen-derived compounds selected for the study differed in chemical structure, which would explain the differences in BBB permeation.

## 4. Materials and Methods

### 4.1. Plant Material

The examined lichens were manually collected: *C. uncialis*, Jastrzębsko Stare, Greater, VI 2015, Poland, *E. prunastri* from the maple bark, West Pomeranian, XI 2015, *P. sulcata*, Podlesice, Silesian region, VIII 2015, Poland, and authenticated by Dr Daria Zarabska-Bożejewicz (The Institute for Agricultural and Forest Environment of the Polish Academy of Sciences in Poznan). Voucher specimens (CUES 2015.06; EPES 2015.11; PSES 2015.08) have been deposited in the herbarium of the Department of Pharmacognosy at Poznan University of Medical Sciences.

### 4.2. Solvents and Chemicals

Formic acid, sodium carbonate, sodium hydroxide, DMSO, acetone, ammonium acetate, copper (II) chloride were purchased from Avantor Performance Materials Poland S.A. (Gliwice, Poland). The Folin–Ciocalteu phenol reagent was from Merck (Darmstadt, Germany). HPLC grade water, HPLC grade acetonitrile, acetate buffer were from JT Baker–Avantor Performance Materials B.V. (Deventer, The Netherlands), tannic acid from Roth GmbH (Karlsruhe, Germany). Salazinic acid, evernic acid, and (−)-usnic acid were isolated and identified in the Department of Pharmacognosy of Poznan University of Medical Sciences. Atranorin was purchased from ChromaDex ((Los Angeles, CA, USA). All other chemicals were from the Sigma–Aldrich Chemical Co. (Taufkirchen, Germany).

### 4.3. Preparation of Extract 

Dried, cleaned and fragmented thalli of *P. sulcata*, *E. prunastri, C. uncialis* (5.0 g) were sonicated at 35 °C for 6 × 30 min with acetone (100 mL × 6) in an ultrasonic bath. The extracts were filtered using Whatman filterpaper No. 1 and concentrated by evaporation using a rotary evaporator under vacuum at 35–40 °C to afford a solid residue (*P. sulcata* 435 mg, with yield of 8.71%; *E. prunastri* 429 mg, with yield of 8.24%; *C. uncialis* 71.52 mg, with yield of 1.43%). 

### 4.4. HPLC Analysis 

Analysis was performed on (Thermo Scientific UltiMate 3000 UHPLC, Waltham, MA USA) system. The separation was achieved on Kinetex C18 column (100 × 2.1 mm, 5 μm) with mobile phase consisting of acetonitrile and 0.5 % formic acid with a flow rate of 0.3 mL/min. The gradient elution started from 5% of acetonitrile to 100% during 10 min. After that step, isocratic elution with 100% acetonitrile proceeded for 2 min. During the final 5 min, the concentration of acetonitrile decreased to the initial condition (5%). The detection wavelength was 254 nm, and the temperature was 40 °C. The method was validated for salazinic acid, evernic acid, and (−)-usnic acid [23].

### 4.5. Total Phenolic Content (TPC) 

TPC was determined using the Folin–Ciocalteu method [71]. 0.1 mL of DMSO/acetone extracts from *P. sulcata*, *E. prunastri, C. uncialis,* prepared at concentrations of 10 mg/mL, 2 mg/mL, and 5 mg/mL, respectively, were mixed with 4.0 mL of distilled water and with 0.5 mL of Folin–Ciocalteu′s reagent. Immediately afterwards, 20% sodium carbonate was added (2.0 mL), and subsequently, the samples were supplemented with distilled water (a total volume of 10 mL). The samples were incubated for 30 min at room temperature (in the dark). The absorbance was measured at 760 nm (spectrophotometer UV/VIS, Lambda 35, Elmer–Perkin, Waltham, MA, USA). The blank contained the DMSO instead of the examined sample. The results were presented as mg of gallic acid equivalent (GAE) per g of a dry extract ± SEM (to prepare the calibration curve of gallic acid, 0.2–0.8 mg/mL concentrations of gallic acid were used).

### 4.6. Determination of Cytotoxicity of Lichen-Derived Substances 

#### 4.6.1. Compounds/Extracts

Stock solutions in dimethylsulfoxide DMSO (Sigma-Aldrich, St. Louis, MO, USA), (10 mM for compounds and 10 mg/mL for extracts) were prepared and stored at −20 °C. For the experiments, the stock solutions were diluted ex tempore to the final selected concentration with complete cell culture medium.

#### 4.6.2. Cell Culture and Assessment of Cell Viability

Human glioblastoma A-172 and T98G cell lines were purchased from the American Type Culture Collection (ATCC, Manassas, VA, USA), and the European Collection of Authenticated Cell Cultures (ECACC, Salisbury, UK), respectively. The cells were grown at 37 °C in 95% humidified and 5% CO_2_ atmosphere. Media recommended by the provider were used to cultivate the cells: ATCC-formulated Dulbecco’s modified Eagle’s medium (DMEM) (Sigma Aldrich, St. Louis, MO, USA), and ATCC-formulated Eagle’s Minimum Essential Medium (EMEM) (Sigma-Aldrich, St. Louis, MO, USA) were used for A-172 and T98G cells, respectively. Moreover, the media were supplemented with 10% fetal bovine serum (FBS) (Biowest, France) and 1% antibiotics (penicillin and streptomycin) solution (Sigma-Aldrich, St. Louis, MO, USA). The medium for the T98G cell line was additionally supplemented with 2 mM glutamine, 1% non-essential amino acids, and 1% sodium pyruvate (all obtained from Sigma Aldrich, St. Louis, MO, USA). For the experiments, the amount of FBS was reduced to 5%. All experiments were carried out 24 h after the cells were seeded on 96-well plates.

The effect of the tested compounds and extracts on GBM cell viability was assessed by measuring the ability of cells to metabolize 3-(4,5-dimethylthiazol-2-yl)-2,5-diphenyl-tetrazolium bromide (MTT), as previously described [72]. In brief, 1 × 10^4^ cells per well were seeded on 96-well plates. After 24 h incubation, they were treated with varying concentrations of lichen-derived compounds (1, 5, 10, 25, 50 and 100 µM) and extracts (1, 5, 10, 25, 50 and 100 µg/mL). Cells treated with medium containing the respective concentrations of DMSO (Sigma-Aldrich, St. Louis, MO, USA) were used as a control. After 48 h incubation the cells were washed with phosphate-buffered saline (PBS) buffer and incubated for 4 h in the presence of PBS containing 0.5 mg/mL MTT salt (Merck, Darmstadt, Germany). Next, the formazan crystals were dissolved in acidic isopropanol. In order to enhance dissolution of formazan crystals the plates were shaken on the orbital shaker for 30 min. Finally, the absorbance was measured at λ = 570 nm and λ = 690 nm on the microplate reader (Tecan Infinite M200). All the experiments were repeated three times with at least four measurements per assay.

### 4.7. Inhibition of Indoleamine 2,3-Dioxygenase (IDO1)

Inhibitory effects of the investigated extracts and standards (salazinic acid, evernic acid, (−)-usnic acid), as well as the reference IDO1 inhibitor (epacadostat) were determined using Universal IDO1/IDO2/TDO Inhibitor Screening Assay Kit from BPS Bioscience, Inc. (San Diego, CA, USA). This colorimetric assay is based on the measurement of the ability of IDO1, IDO2 and TDO to convert l-tryptophan into *N*-formylkynurenine (NFK). The experiments were performed according to the manufacturer’s guidelines. The final concentration of the compounds and extracts in the reaction mixture was 100 µg/mL. The amount of NFK was measured spectrophotometrically at 320 nm using an Epoch BioTek microplate reader (BioTek Instruments, Inc., Winooski, VT, USA). The samples were run in triplicate and the results were expressed as mean ± SEM.

### 4.8. Effect on Cyclooxygenase-2 (COX-2) Activity

For the assay, reagents from Cayman COX Activity Assay Kit (Chemical, Ann Arbor, MI, USA, No. 760151) were prepared strictly as suggested by the producer and were combined with COX-2 enzyme (Human recombinant, Cayman No. 60122, pre-diluted 100-fold using 100 mM, pH 8.0 Tris buffer). A volume of 0.01 mL of the studied sample, dissolved in pure DMSO to obtain 5 mg/mL, was mixed with 0.12 mL of Tris buffer (100 mM, pH 8.0), 0.01 mL hemin, shaken and left for 5 min at 25 °C followed by addition of 0.02 mL colorimetric substrate and 0.02 mL arachidonic acid solution. To start the reaction, 0.02 mL of COX-2 solution was added. The increase of absorbance during incubation at room temperature was recorded at 590 nm. Negative (blank) sample (buffer instead of studied sample) and positive sample (COX-2 inhibitor DuP-697) were run simultaneously. Background of studied samples (0.01 mL of sample mixed with 0.19 mL buffer) was also measured and included in the calculations. Each sample was run in at least 4 repeats. Inhibition of the enzyme activity was expressed in % (indicates by how many % the activity has been reduced in relation to the negative or blank sample for which the maximum activity was assumed as 100%, under the conditions used in the method). Also, inhibition of enzyme activity was expressed as acetylsalicylic acid equivalent concentration (mg/mL). For this purpose, acetylsalicylic acid solutions were prepared at 14 concentrations (0.2-10.0 mg/mL) and analyzed similarly to tested samples.

### 4.9. Anti-Hyaluronidase Activity 

Inhibition of hyaluronidase (HA) was determined by a method described by Studzińska-Sroka et al. [23]. Briefly, 25 µL of incubation buffer (50 mM, pH 7.0, with 77 mM NaCl and 1 mg/mL of albumin), 25 µL of enzyme (30 U/mL of acetate buffer pH 7.0), 10 µL solutions of the tested extracts (1.25–7.5 mg/mL) or lichen substances (0.625–5.0 mg/mL), and 15 µL of acetate buffer (pH 4.5) were mixed (the final concentrations were: 0.125–0.75 mg/mL). After incubation at 37 °C for 15 min, 25 µL of HA (0.3 mg/mL in acetate buffer) was added. Subsequently, the plate was incubated for 45 min (37 °C). After this time, 200 µL of 2.5% CTAB in 2% NaOH was put in. The turbidance of the reaction mixture was measured as the absorbance at 600 nm (Multiskan GO 1510, Thermo Fisher Scientific, Vantaa, Finland) after 10 min of incubation at room temperature. β-escin was used as the positive control (6.0–10.0 mg/mL, with the final concentration 0.6–1.0 mg/mL). All experiments were carried out three times and the average from *n* = 5 (lichen substances) or *n* = 6 (lichen extracts and β-escin) measurements was calculated. The percentage of inhibition was calculated by using the equation below.

(1) $$\%\;\mathrm{inhibition}\;\mathrm{activity}=\frac{\left(T_{s}-{\mathrm{TE}}_{\mathrm{blank}}\right)}{\left({\mathrm{TH}}_{\mathrm{blank}}-{\mathrm{TE}}_{\mathrm{blank}}\right)}\times 100\%$$

where: T_S_ = absorbance of sample; TE_blank_ = absorbance of the enzyme + examined substance; TH_blank_ = absorbance of the HA + examined substance.

### 4.10. Antioxidant Activity 

#### 4.10.1. DPPH and CUPRAC analysis

Two methods were used to test the antioxidant activity: DPPH and CUPRAC. The DPPH assay was effected according to Kikowska et al. (2018) [73], with slight modifications. Briefly, 25.0 μL of examined extracts or compounds were prepared at different concentrations (within the range 1.25–20 mg/mL, for each extract, and with two different concentrations: 3 mg/mL, and 6 mg/mL, for salazinic acid, evernic acid and (−)-usnic acid). Each was mixed with 175.0 μL of DPPH^•^ solution (3.9 mg DPPH in 50.0 mL of MeOH); the reached final assay concentrations were: 156.25 μg/mL to 2500 μg/mL, for extracts, and 375 μg/mLto 750 μg/mL, for lichen compounds. The samples were then shaken and incubated in the dark (30 min) at room temperature. Next, the absorbance was measured at 517 nm. The control blank contained 25.0 μL of DMSO and 175.0 μL of DPPH^•^ solution. For calculating the scavenging % of DPPH^•^ free radicals, the following formula was used:

DPPH scavenging activity (%) = [(A_0-_A_1_)/A_0_] × 100%, (2)

where A_0_ is the absorbance of the control and A_1_ is the absorbance of the sample. The IC_50_ values, i.e., (a concentration of antioxidant necessary to halve the initial DPPH^•^ quantity), were used to compare the quality of the antioxidant potency of the studied extracts. The lower absorbance of the reaction mixture indicated a higher free radical scavenging activity. For the investigated substances, two independent experiments were carried out and the average from *n* = 3 measurements was calculated.

To measure the antioxidant capacity of the lichen extracts and compounds the CUPRAC assay was used [23]. The CUPRAC reagent was freshly prepared before the analysis and composed of equal parts of acetate buffer (pH 7.0), 7.5 mM neocuproine (Sigma-Aldrich, St. Louis, MO, USA) solution in 96% ethanol, and 10 mM CuCl_2_xH_2_O solution. The samples (50 μL) dissolved in DMSO at different concentrations (78 μg/mL–1250 μg/mL, for extracts, and 125–1000 μg/mL, for compounds), were mixed with the CUPRAC reagent (150 μL) (the final assay concentrations were: 19 μg/mL – 312 μg/mL, for extracts, and 31 μg/mL–250 μg/mL, for compounds). After shaking and incubating in the dark at room temperature for 30 min, the absorbance was read at 450 nm. Resveratrol was used as a standard (25–400 μg/mL; the final assay concentrations were 6.25–100 μg/mL). The results were expressed as the IC_0.5_, the concentration at which the absorbance was 0.5. For the investigated substances, two independent experiments were carried out and the average from *n* = 3 measurements was calculated.

#### 4.10.2. Effect on Antioxidant Enzymes Activity

##### Effect on Superoxide Dismutase Activity (SOD)

Sample (50 µL, 5 mg/mL of pure DMSO) was mixed with 10 μL SOD (0.24 U), 160 μL nitrobluetatrazolium solution (0.0025 M), 205 μLphosphate buffer (0.2 M, pH 7.5), 30 μL xanthine (150 mM in 1 M NaOH) and 0.01 mL xanthine oxidase (0.065 U, Sigma Aldrich X4875). The change in the absorbance at 550 nm was measured in tested samples *vs.* controls without the studied sample after 20 min of incubation and the effect on the enzyme was calculated using the equation [74]:

(3) $$\mathrm{Inhibition}\;\left[\%\right]=100\;–\;100\times \frac{\left(\mathrm{Abs}.\;30\;\min\;–\;\mathrm{Abs}.\;0\;\min\right)}{\left(\mathrm{Abs}.\;\mathrm{control}.\;30\;\min\;–\;\mathrm{Abs}.\;\mathrm{control}\;0\;\min\right)}$$

##### Effect on Glutathione Reductase (GR) Activity

Sample (20 µL, 5 mg/mL of pure DMSO) was mixed with 10 μL of EDTA solution and 12 μL of GSSG solution and incubated for 5 min at 25 °C; 4 μL of NADPH solution and was then added (all reagents were dissolved in 0.1 mM sodium phosphate buffer, pH 7.6), and the initial absorbance (340 nm) was recorded. The reaction was then started by addition of 2 U glutathione reductase (2 μL, Sigma Aldrich no G3664), 177 μL of 0.1 mM sodium phosphate buffer, and the absorbance was recorded after 5 min of incubation at 25 °C. Concentrations of reagents in the final mixture (805 μL) were as follows: 0.5 mM EDTA, 10 mM GSSG and 10 mM NADPH. Blank sample was prepared with buffer instead of the sample and background was measured (mixture containing studied sample and buffer only). One unit of enzyme activity has been defined as nMol of NADPH consumed/min·mL sample, in comparison with nMol of NADPH consumed/min in blank (reagent) sample [75].

##### Effect on Glutathione Peroxidase (GPx) Activity

Sample (20 µL, 5 mg/mL of pure DMSO) was mixed with 8 μL of EDTA solution, 10 μL of glutathione reductase (0.2 U Sigma G3664), 4 μL of GSH solution, 10 μL of glutathione peroxidase (0.04 U Sigma G6137), 22 μL of H_2_O_2_ and 332 μL of 50 mM sodium phosphate, pH 7.0). To start the reaction, 4 μL of NADPH solution (N5130) was added and the decrease in the absorbance (340 nm) was read after 10 min of incubation at 25 °C. All solutions were prepared in 50 mM buffer and concentrations of reagents in the final mixture were as follows: 1mM EDTA, 0.2 U glutathione reductase, 2 mM GSH, 0.04 U glutathione peroxidase, 1.5 mM H_2_O_2_ and 0.8 mM NADPH. Blank sample was prepared with buffer instead of the studied sample and background was measured (mixture containing studied sample and buffer only). One unit of enzyme activity was defined as nMol of NADPH consumed/min·mL sample, in comparison with nMol of NADPH consumed/min in blank (reagent) sample [76].

### 4.11. Anti-Cholinesterase Activity

Ellman’s colorimetric method was used [77] with modifications described previously [78]. Tested sample (10 μL) at concentration 5 mg/mL was mixed with 20 μL of AChE (or BChE) solution (0.28 U/mL) and completed after 5 min with 35 μL of ATChI (or BTCh) (1.5 mmol/L), 175 μL of 0.3 mmol/L DTNB (containing 10 mmol/L NaCl and 2 mmol/L MgCl_2_) and 110 μL with Tris-HCl buffer (50 mmol/L, pH 8.0). Samples containing 10 μL of Tris-HCl buffer instead of the studied sample were run in the same way (“blank” samples). The increase in the absorbance due to the spontaneous hydrolysis of the substrate was monitored using “blank” samples containing ATCh (or BTCh) and DTNB completed to 350 μL with Tris-HCl buffer. All samples were incubated at 22 °C (30 min; incubation time was determined after optimization experiments, details not shown), and the absorbance was measured (405 nm, 96-well microplate reader, Tecan Sunrise, Grödig, Austria). The “false-positive” effect of studied compounds was measured according to Rhee et al. [79] with minor modifications, as described previously [78]: after mixing of the substrate with the enzyme and buffer, the “false-positive” sample was left for incubation. Then, a studied sample and DTNB were added, followed by an immediate measurement of the absorbance.

Reference cholinesterase inhibitors were used for the calculations of results (eserine, neostigmine, magniflorine, rivastigmine and donepezil). For this purpose, for each compound, 16 dilutions in pure DMSO were prepared (2.57–41.14 μg/mL). These solutions (10 μL) were tested as described above and calibration curves were produced.

Each sample was analyzed in at least eight repeats, and all solutions used in a set of analyses were prepared in the same buffer. For calculations, the background of the sample (10 μL mixed with 340 μL of Tris buffer) was measured at 405 nm and subtracted during calculations. Then, the absorbance of the test sample was subtracted from the absorbance of the “blank” sample.

### 4.12. Permeability through the Blood-Brain-Barrier (PAMPA-BBB)

To evaluate the effective permeability (*Pe*) of the salazinic acid, evernic acid, (−)-usnic acid as pure compounds and from the extracts (*P. sulcata* acetone extract*, E. prunastri* acetone extract, *C. uncialis* acetone extract, respectively), Parallel Artificial Membrane Permeability Assay (PAMPA) for the Blood-Brain Barrier (BBB) was used (Pion Inc., Billerica, MA, USA). The stock solutions of acetone extracts from *C. uncialis*, *E. prunastri* and *P. sulcata* and from (−)-usnic acid, evernic acid, salazinic acid, were prepared with DMSO (the concentrations of salazinic acid, evernic acid, and (−)-usnic acid reached were 2.5 mg/mL for pure compounds and extracts). Next, the donor solution was prepared (5 µL of stock solution/1000 µL of Prisma buffer, pH = 7.4 (Prisma HT, Pion Inc.). Subsequently, each filter membrane of the acceptor plate wells was coated with 5 μL BBB-1 lipid solution (Pion Inc.), and 180 µL of the donor solution was added to the donor wells. The acceptor well was filled with 200 µL BSB (Brain Sink Buffer, Pion Inc.). The plate was incubated for 1 h or 4 h at 37 °C. After incubation, the donor and acceptor concentrations of examined substances were determined using the HPLC. Effective permeability (*Pe*) of the compounds was calculated by using the following equation.

(4) $$P_{e}=-\frac{\ln\left(1-\frac{C_{A}}{C_{\mathrm{eq}}}\right)}{S\times \left(\frac{1}{V_{D}}+\frac{1}{V_{A}}\right)\times t}$$

where *Pe* is the effective permeability coefficient (cm/s), V_D_ = donor volume, V_A_ = acceptor volume, C_eq_ = equilibrium concentration and $C_{\mathrm{eq}}=\frac{C_{D}\times V_{D}+C_{A}\times V_{A}}{V_{D}+V_{A}}$, S = membrane area, and t = incubation time (in seconds) [35,36].

Compounds with *Pe* (× 10^−6^ cm/s) > 1.5 are classified as high permeation predicted, while *Pe* (× 10^−6^ cm/s) < 1.5 are classified as low permeation predicted. Samples were analyzed in triplicate and the average is reported.

### 4.13. Statistical Analysis

Results were expressed as means ± SEM. The median effect concentrations (IC_50_ or IC_0.5_ values) were determined using a concentration–response curve. Statistical differences were calculated using the unpaired t-test with two-tailed distribution, with significant differences considered at *p* < 0.05.

## 5. Conclusions

To conclude, our study shows that lichen-derived compounds and extracts exert cytotoxic activity against GBM cells, inhibit the enzymes involved in the kynurenine pathway, COX-2, and hyaluronidase, and have very mild antioxidant properties, making them good candidates for adjuvant anti-GBM therapeutics. Usnic acid, with its ability to cross the BBB and reduce GBM cell proliferation, can be regarded as a prototype for compounds with activity within the CNS, in particular for GBM treatment. Lichen-derived compounds and extracts should also be further evaluated as neuroprotective agents.

## Figures and Tables

**Table 1:** The IC_50_ of the analyzed lichen-derived compounds/extracts established after 48 h treatment of A-172 and T98G cell lines.

Lichen-Derived Compound/ Extract	A-172		T98G	
	IC_50_ (µg/mL)	IC_50_ (µM)	IC_50_ (µg/mL)	IC_50_ (µM)
Salazinic acid	>38.8	>100.0	>38.8	>100.0
*P. sulcata* extract	73.6 ± 7.3	-	89.8 ± 5.1	-
Evernic acid	>33.2	>100.0	>33.2	>100.0
*E. prunastri* extract	73.8 ± 5.5	-	61.0 ± 1.1	-
(−)-Usnic acid	31.5 ± 0.8	91.4 ± 2.0	13.0 ± 1.3	37.8 ± 3.8
*C. uncialis* extract	11.0 ± 4.6	-	3.9 ± 2.2	-

IC_50_ ± SEM was calculated from the results obtained in three independent experiments with four measurements per assay, for each point in the concentration curve.

**Table 2:** Effect of the investigated lichen extracts and reference compounds on the activity of indoleamine-2,3-dioxygenase (IDO1, IDO2) and tryptophan-2,3-dioxygenase (TDO).

Lichen-Derived Compound/Extract	Inhibition (%)		
	IDO1	IDO2	TDO
Salazinic acid	-	-	-
*P. sulcata* extract	20.5 ± 1.2	-	-
Evernic acid	32.8 ± 1.9	-	-
*E. prunastri* extract	43.1 ± 2.0	-	-
(−)-Usnic acid	21.6 ± 0.9	-	-
*C. uncialis* extract	54.8 ± 3.5	-	-
epacadostat	95.6 ± 2.8	-	-

“-“—not active (i.e., inhibitory effect was lower than 10%). Concentration of examined samples was 100 µg/mL. The mean values ± SEM from three independent measurements are presented.

**Table 3:** Inhibition of cyclooxygenase-2 (COX-2) enzyme by the extracts of *P. sulcata*, *E. prunastri*, and *C. uncialis* as well as their major secondary metabolites: salazinic acid, evernic acid and (−)-usnic acid.

Lichen-Derived Compound/Extract	Equivalent Concentration of Acetylsalicylic Acid (mg/mL)	COX-2 Inhibition (%)
Salazinic acid	12.9 ± 0.1	60.3 ± 3.0
*P. sulcata* extract	13.0 ± 0.8	65.9 ± 4.1
Evernic acid	10.9 ± 1.8	50.7 ± 2.1
*E. prunastri* extract	10.0 ± 0.2	35.9 ± 2.8
(−)-Usnic acid	12.9 ± 1.5	59.3 ± 3.5
*C. uncialis* extract	10.4 ± 0.1	45.9 ± 1.9

Concentration of examined samples: see Section 4. The mean values ± SEM from three independent experiments with four measurements per assay are presented.

**Table 4:** Antioxidant activity of lichen extracts and compounds measured using DPPH and CUPRAC analysis.

Lichen-Derived Compound/Extract	DPPH IC_50_ (µg/mL)	CUPRAC IC_0.5_ (µg/mL)
Salazinic acid	>750.0	>250.0
*P. sulcata* extract	669.3 ± 11.8	175.4 ± 1.0
Evernic acid	>750.0	>250.0
*E. prunastri* extract	1926.3 ± 33.2	103.4 ± 1.4
(−)-Usnic acid	>750.0	>250.0
*C. uncialis* extract	>2500.0	>312.5
resveratrol	25.1 ± 0.1	29.7 ± 0.1

IC_50_—the IC_50_ values, i.e., the concentration of an antioxidant necessary to halve the initial DPPH^•^ concentration (for lichen-derived substances the highest concentration was 750.0 µg/mL, for extracts 2500.0 µg/mL); IC_0.5_ values, i.e., the concentration of an antioxidant necessary to achieve the absorbance of 0.5 in the CUPRAC analysis (for lichen-derived substances the highest concentration was 250.0 µg/mL, for extracts 312.5 µg/mL). The mean values ± SEM from three measurements are presented (*n* = 3).

**Table 5:** Effect of lichen extracts and compounds on SOD activity.

Lichen-Derived Compound/Extract	SOD Inhibition (%)
Salazinic acid	19.4 ± 0.3
*P. sulcata* extract	22.0 ± 1.3
Evernic acid	21.2 ± 0.0
*E. prunastri* extract	53.4 ± 2.4
(−)-Usnic acid	19.6 ± 0.6
*C. uncialis* extract	12.8 ± 1.0

Concentration of examined sample in the reaction mixture: 537.6 μg/mL reaction mixture. The mean values ± SEM from three measurements are presented (*n* = 3).

**Table 6:** Effect of lichen extracts and compounds on GR and GPx activity.

Lichen-Derived Compound/Extract	GR Inhibition under Reaction Conditions (%)	GR Inhibitory Activity (nMol Depleted NADPH/min Incubation)	GPx Inhibition under Reaction Conditions (%)	GPx Inhibitory Activity (nMol Depleted NADPH/min Incubation)
Salazinic acid	-	-	-	-
*P. sulcata* extract	-	-	47.1 ± 0.7	93.9 ± 5.2
Evernic acid	-	-	20.0 ± 2.1	39.9 ± 1.0
*E. prunastri* extract	91.1 ± 7.2	3551.2 ± 264.4	92.4 ± 4.3	184.2 ± 30.2
(−)-Usnic acid	18.2 ± 2.2	710.2 ± 42.3	13.7 ± 2.3	27.3 ± 1.3
*C. uncialis* extract	-	-	-	-

“-“—not active; concentration of examined sample in the reaction mixture: 444.4 μg/mL (GR), 243.9 μg/mL (GPx).

**Table 7:** Inhibition of AChE and BChE by lichen substances expressed as equivalent reference concentration.

Lichen-Derived Compound/Extract	Equivalent Reference Concentration (μg/mL)									
	Neostygmine		Magniflorine		Donepezil		Eserine		Rivastigmine	
	AChE	BChE	AChE	BChE	AChE	BChE	AChE	BChE	AChE	BChE
Salazinic acid	-	13.6 ± 0.1	-	43.2 ± 0.2	-	7.9 ± 0.3	-	9.0 ± 0.1	-	70.9 ± 0.3
*P. sulacata* extract	-	8.1 ±0.0	-	26.2 ± 0.6	-	4.8 ± 0.1	-	5.4 ± 0.6	-	42.9 ± 0.1
Evernic acid	-	16.5±0.2	-	52.3 ± 0.1	-	9.5 ± 0.0	-	10.9 ± 0.1	-	85.9 ± 0.1
*E. prunastri* extract	-	-	-	-	-	-	-	-	-	-
(−)-Usnic acid	-	-	-	-	-	-	-	-	-	-
*C. uncialis* extract	0.7 ± 0.1	16.5 ± 0.1	7.9 ± 0.0	52.3 ± 0.1	0.6 ± 0.1	9.5 ± 0.4	0.1 ± 0.0	10.9 ± 0.0	1.4 ± 0.0	85.9 ± 0.2

“-“—not active; concentration in the reaction mixture: 142.9 μg/mL.

**Table 8:** The effective permeability (*Pe*) of salazinic acid, evernic acid, (−)-usnic acid as pure compounds and from extracts using the Parallel Artificial Membrane Permeability Assay for the Blood-Brain Barrier (PAMPA-BBB).

Lichen-Derived Compound/Extract	*Pe* × 10^−6^ (cm/s) t = 1 h	*Pe* × 10^−6^ (cm/s) t = 4 h
Salazinic acid (PC)	np	np
Salazinic acid (from PSE)	np	np
Evernic acid (PC)	5.2 ± 0.8	8.6 ± 0.4
Evernic acid (from EPE)	5.0 ± 0.7	7.2 ± 0.4
(−)-Usnic acid (PC)	92.8 ± 6.3	nd
(−)-Usnic acid (from CUE)	140.5 ± 7.3	nd

Np—classified as non-permeable (i.e., *Pe* < 0.5 × 10^−6^ cm/s); nd – not determined; PC—pure compound; PSE—*P. sulcata* acetone extract; EPE—*E. prunastri* acetone extract; CUE—*C. uncialis* acetone extract. Results are presented as *Pe* × 10^−6^ cm/s. The mean values ± SEM from three independent experiments are presented (*n* = 3).

## Data Availability

Data is contained within the article.
